# Impact of Canopy Openness on Spider Communities: Implications for Conservation Management of Formerly Coppiced Oak Forests

**DOI:** 10.1371/journal.pone.0148585

**Published:** 2016-02-04

**Authors:** Ondřej Košulič, Radek Michalko, Vladimír Hula

**Affiliations:** 1 Department of Forest Protection and Wildlife Management, Faculty of Forestry and Wood Technology, Mendel University in Brno, Brno, Czech Republic; 2 Department of Forest Ecology, Faculty of Forestry and Wood Technology, Mendel University in Brno, Brno, Czech Republic; 3 Department of Botany and Zoology, Faculty of Science, Masaryk University, Brno, Czech Republic; 4 Department of Zoology, Fisheries, Hydrobiology and Apiculture, Faculty of Agronomy, Mendel University in Brno, Brno, Czech Republic; Charles University in Prague, CZECH REPUBLIC

## Abstract

Traditional woodland management created a mosaic of differently aged patches providing favorable conditions for a variety of arthropods. After abandonment of historical ownership patterns and traditional management and the deliberate transformation to high forest after World War II, large forest areas became darker and more homogeneous. This had significant negative consequences for biodiversity. An important question is whether even small-scale habitat structures maintained by different levels of canopy openness in abandoned coppiced forest may constitute conditions suitable for forest as well as open habitat specialists. We investigated the effect of canopy openness in former traditionally coppiced woodlands on the species richness, functional diversity, activity density, conservation value, and degree of rareness of epigeic spiders. In each of the eight studied locations, 60-m-long transect was established consisting of five pitfall traps placed at regular 15 m intervals along the gradient. Spiders were collected from May to July 2012. We recorded 90 spider species, including high proportions of xeric specialists (40%) and red-listed threatened species (26%). The peaks of conservation indicators, as well as spider community abundance, were shifted toward more open canopies. On the other hand, functional diversity peaked at more closed canopies followed by a rapid decrease with increasing canopy openness. Species richness was highest in the middle of the canopy openness gradient, suggesting an ecotone effect. Ordinations revealed that species of conservation concern tended to be associated with sparse and partly opened canopy. The results show that the various components of biodiversity peaked at different levels of canopy openness. Therefore, the restoration and suitable forest management of such conditions will retain important diversification of habitats in formerly coppiced oak forest stands. We indicate that permanent presence of small-scale improvements could be suitable conservation tools to prevent the general decline of woodland biodiversity in the intensified landscape of Central Europe.

## Introduction

Lowland woodlands dominated by oaks are among the important land-use types due to their production and non-production functions and cover around 10% of the total forest area in the Czech Republic [1–2]. However, they recently challenge to overal biodiversity loss due to large-scale unification characterized by dark and undisturbed conditions or conversely by wholly open and disturbed canopy coverage [2]. This is in contrast to these forests’ past state, which had been marked by far more dynamic and diversified conditions [2]. Before conversion of most coppiced forests to high forests, lowland oak woodlands had to satisfy a variety of human demands. They had been used as coppice forests, as pasture, and for pollarding to produce litter material and firewood [3–5]. Traditionally managed oak woodlands were open, sunny, and with diverse mosaics of succession development, thus resulting in high biodiversity [2,6]. Many associated organisms, including thermophilous species of numerous plants, fungi, and animals had colonized these habitats maintained by old styles of forest managements existing from the Mediterranean to Northern Europe [4–9].

Coppicing was among the most widespread traditional management styles for lowland broadleaved woodlands across all of Europe [4]. In coppiced forests, trees were usually cut down every 5–20 years in order to regenerate sprouting from stumps. This provided a cyclical pattern of extreme changes in ground-level light penetration [4,10]. This forest management in combination with traditional small-scale land ownership and rapid wood harvest rotations produced many varied habitat structures, ranging from dense and shady conditions to sparse and open places, accompanied by great diversity of forest species as well as of xeric specialized invertebrates [11]. Coppicing and other traditional forestry methods (e.g., woodland pasture, litter harvesting) rapidly declined during the 20th century, however, due to the political and economic changes in Central and Eastern Europe after the Second World War [7,8]. Coppiced broadleaved oak forests were taken out of private ownership, abandoned, and later transferred to state holdings, thereby producing unified, large blocks of high forests primarily for timber production. Furthermore, some areas of formerly coppiced woodlands were conserved as protected areas without any active management in order to return them to a more natural, undisturbed state, which formerly was regarded as beneficial for biodiversity and nature protection [12,13]. During the ensuing several decades, however, once originally heterogeneous landscape made up of open-and-sparse woodlands and forest-steppes developed into much more uniform and closed forest areas. These land-use changes have resulted in drastic reduction of the landscape’s overall biodiversity, thus most likely causing a sizeable extinction debt [14,15]. In particular, the populations and local species richness of arthropods favoring open and sparse woodlands have diminished greatly [2, 4, 16,17].

The negative impact of abandoning traditional forestry methods and the ensuing woodland canopy closure has been well documented by numerous studies investigating such various taxa as higher plants [9,18–19], butterflies [20–22], saproxylic beetles [23–25], Diptera and Hemiptera [26], and birds [27]. Concerning spiders, which constitute a significant part of forest ecosystems, we are aware of just one study focused upon the impact of game management on spider diversity in one small area of coppiced forests in the Czech Republic [11]. Collectively, all of those authors cited above propose a return to such traditional management as coppicing, which forms a dynamic mosaic of various microhabitats supporting high species diversity in woodlands. Despite these important findings, coppicing remains a neglected type of forest management in most European countries and occupies an area of considerably less than 1% of all woodlands [28]. The reasons for this include contrary forestry policies, low economical efficiency and very often uncooperative conservationists [17]. Recommendations for active woodland management are often easily rejected due to the nonintervention policy in protected areas and the argument that management favors only certain components of biodiversity and may threaten other groups of organisms [11]. Therefore, detailed knowledge as to the influence of different habitat structures on species composition and biodiversity is needed before promoting the sustainable management of lowland oak woodlands.

The canopy openness gradient can be used to assess the relationship among different components of biodiversity and potential forest management. It utilizes the precise level of light, which is a major determinant in different habitat structures [29,30]. This is a unique tool and simulates conditions according to different management interventions that influence the openness or closure of forest habitats which contribute to changes in several biodiversity components [31–34]. Such an evaluation also can show the distribution pattern of useful bioindicator organisms along the vegetation structure and light volume gradient in broadleaved forests [30].

Spiders (Araneae) constitute ideal bioindicator organisms for determining the effect of the canopy openness gradient on biodiversity conservation in such lowland oak woodlands as formerly coppiced stands. In forests, spiders intensively react to such microhabitat conditions as light, moisture, temperature, vegetation structure, and litter floor while landscape features are less important [35–41]. Moreover, spiders are very abundant in forest ecosystems, being present from litter layers to the canopy, and they are mostly generalist predators [37,38,42]. As such, they significantly affect the dynamics of forest food webs through cascading effects [43–45]. Spiders have evolved numerous predatory strategies resulting in many different functional roles that also enable the precise evaluation of functional diversity [46,47].

The objective of the present study was to investigate the impact of canopy openness on epigeal spider assemblages in abandoned coppice forests in order to develop conservation recommendations for forest management in lowland woodlands. In particular, we studied the effect of canopy openness on species richness, activity density, functional diversity, community composition, conservation value, and degree of rareness. We hypothesized that even the small-scale habitat structures predicted by different levels of canopy openness in transferred coppiced forest may create conditions suitable for spider diversity generally, as well as for species of specific conservation concern, through their heterogeneous mosaics of microhabitat diversifications. In this manner, they may increase the overall landscape biodiversity in response to forest and conservation management.

## Materials and Methods

### Research area and study sites

**Fig 1:**
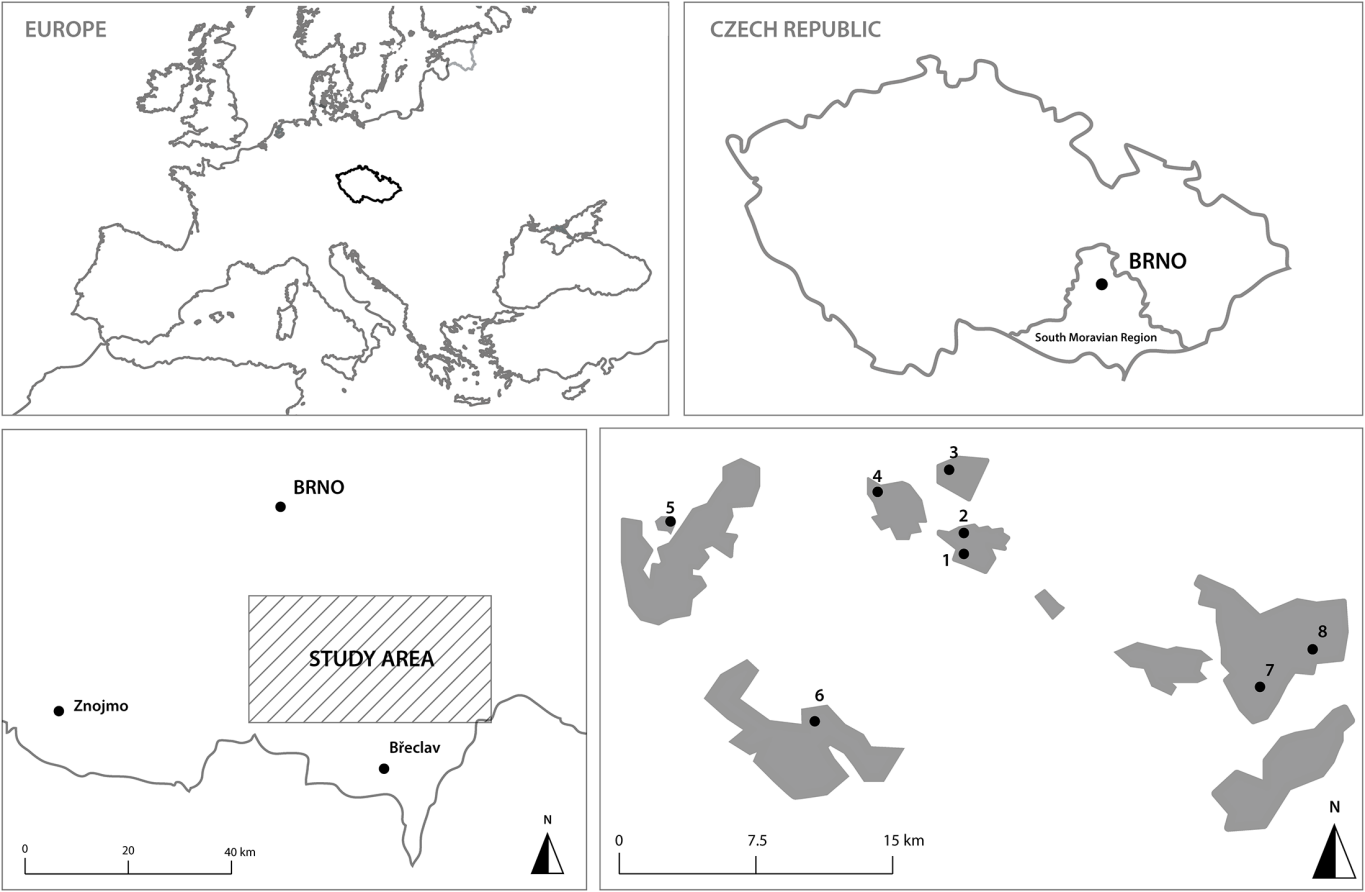
Map of the research area in the south-eastern Czech Republic with a local map showing forest (grey), non forest areas (white) and exact localization of study sites. See Table 1 for locality identification according to numbering. Map background was designed by Freepik as a shareware of vector maps for free using and downloaded from http://www.freepik.com/. Forest patches (grey color) were used according to official forest maps of Forest Management Institute (http://www.uhul.cz/home) and by Faculty of Forestry and Wood Technology, Mendel University in Brno (author´s institution)

The study was performed in the South Moravian Region of the Czech Republic, within the Brno, Břeclav, and Hodonín districts, situated close to the borders with Austria and Slovakia (Fig 1). This area is a crossroads between the Hercynian highlands, the Carpathians, and the Pannonian biogeographic region, and it hosts a great biodiversity of thermophilic fauna and flora [48]. It encompasses around 650 km^2^ in a lowland type of landscape (150–350 m a.s.l.) that consists of a mosaic of intensified arable land, settlements, deciduous forests, orchards, and small patches of open grasslands [48]. The climate is warm and relatively dry, with average annual temperature around 9.2°C and average annual precipitation near 550 mm [49].

Until the mid-20th century, a major part of the present forest area (with the exception of most of the floodplain forests) in the South Moravian Region was coppiced and subject to such other traditional management techniques as pasturing and litter harvesting [7,11,48]. Recently, these forests were partly transferred to nature conservation areas and game management areas or they were used as older-aged commercial forests. In general, a strong shift from species-rich heterogeneous woodlands toward species-poorer communities was observed due to the canopy closure after the abandonment of coppicing [8,9].

We selected eight main and largest formerly coppiced forests in the major woodland area of the South Moravian Region in the municipalities of Morkůvky, Němčičky, Boleradice, Kurdějov, Vranovice, Milovice, Dubňany, and Hodonín (Fig 1, Table 1). All study locations were afforested with various species of oaks (*Quercus robur*, *Q*. *pubescens*, *Q*. *petraea*,) and hornbeam (*Carpinus betulus*) as the main tree species. Accompanying tree species included ash (*Fraxinus excelsior*), linden (*Tilia cordata*), and field maple (*Acer campestre*). Phytocenological units were consistent across study locations and characterized by *Q*. *pubescens–Q*. *petraea* plant communities. All study sites had similar altitudes and comparable forest growth ages (>80 years) and are now managed in large scale as high-stand forests (some locations were sequentially thinned to forest clearings in small spatial patches to open the canopy cover; at two locations, Milovice and Jesličky, were used conservation managements aimed to the opening of forest edges). Table 1 characterizes the particular study sites and includes species-richness records.

**Table 1:** Characteristics of individual study sites located across South Moravia in the Czech Republic.

No.	Location	District	Coordinates	Altitude (m a.s.l.)	Species^a^	Red-listed species^b^	Rare specialized species^c^
1	Němčičky	Břeclav	48°56′35′′N,	245	41	11	15
			16°50′19′′E				
2	Morkůvky	Břeclav	48°57′12′′N,	280	36	10	16
			16°50′11′′E				
3	Boleradice	Břeclav	48°56′43′′N,	315	28	1	7
			16°50′55′′E				
4	Kurdějov	Břeclav	48°58′58′′N,	298	23	4	6
			16°46′41′′E				
5	Vranovice	Brno	48°57′25N,	202	30	5	7
			16°35′46′′E				
6	Milovice	Břeclav	48°50′55′′N,	225	32	9	13
			16°41′34′′E				
7	Mutěnice	Hodonín	48°52′51′′N,	180	40	14	16
			17°42′49′′E				
8	Dubňany	Hodonín	48°53′30′′N,	204	31	8	13
			17° 7′10′′E				

^a^Total number of species found.

^b^Total number of species included in the national Red List [61].

^c^Total number of species specialized for xeric open forest-steppe and sparse oak forests [51].

### Sampling design

Pitfall traps were used to sample epigeic spiders in the studied forest stands. Each pitfall trap consisted of a plastic cup (500 ml, 9 cm in diameter, 15 cm long) sunk flush with the soil surface. Each trap was filled with a 3–4% solution of formaldehyde and detergent as a killing and fixative fluid.

**Fig 2:**
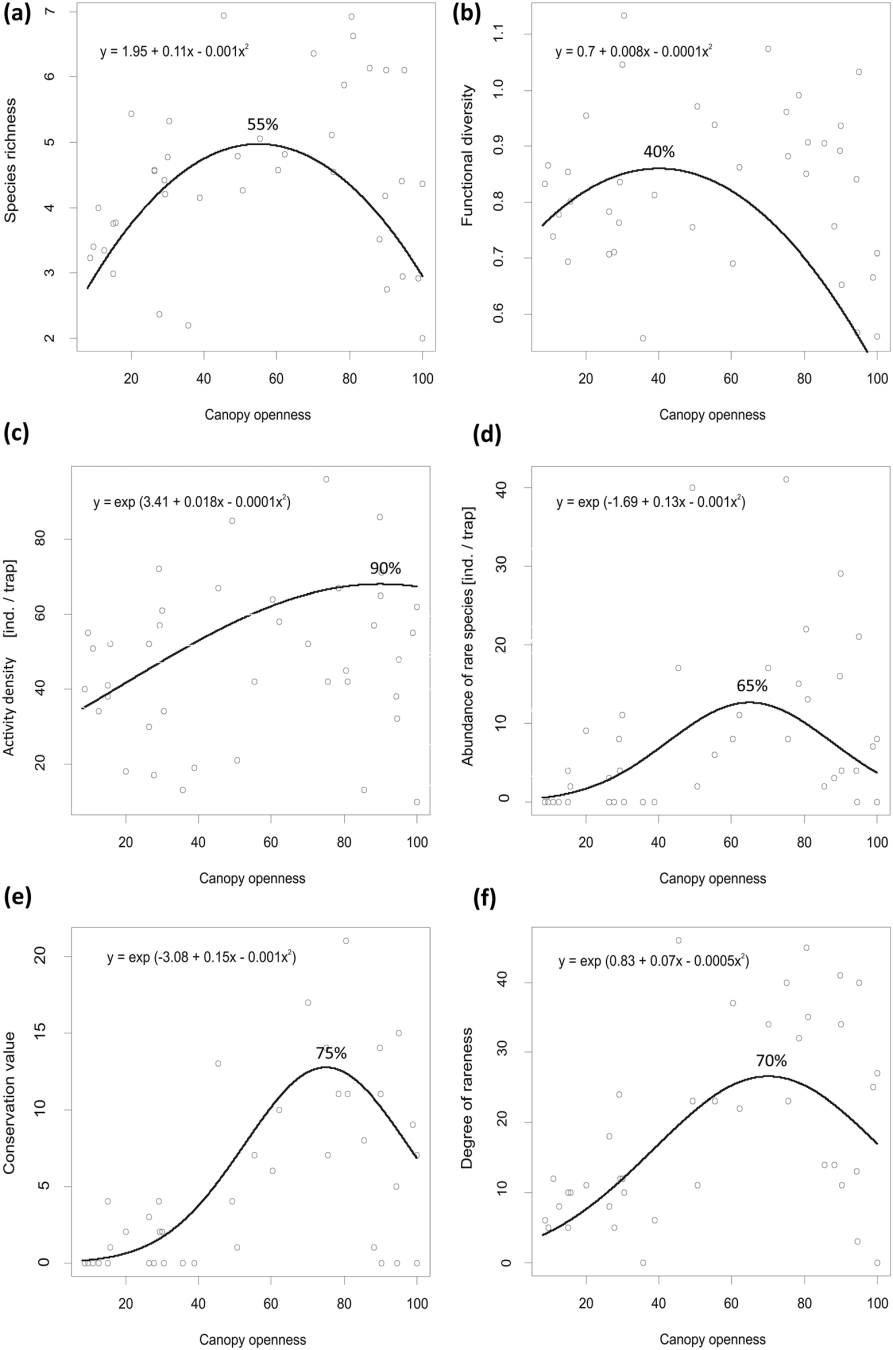
Relationship between canopy openness and diversity indicators of spiders: a) species richness, b) functional diversity, c) activity density, d) abundance of rare and threatened spider species, e) conservation value, f) degree of rareness.

We established transects 60 m long reflecting the canopy openness gradient in each of the eight studied forest stands. Thus, every example of canopy closeness/openness is presented eight times. Transects consisted of five pitfall traps placed at regular 15 m intervals, summing to 40 traps in total. Each trap location presented habitat structure differing in light conditions by canopy openness (Fig 2, in percentage) while ranging from forest clearing (90–98% canopy openness) to dense forest (9–19% canopy openness).

All of the pitfall traps were activated at the 1 May and deactivated at the 20 July 2012. Traps were emptied two times, thus producing a total of 80 samples. Each trap was working for a period of 75 days (i.e., 3,000 days for our trapping design). We assumed that every individual had an equal probability of being captured. This sampling period was chosen because most Central European spiders reach adulthood during this season and can therefore be determined to species level and used for further analyses [50,51]. Juveniles were included only in order to investigate the distribution of spider activity density along the gradient.

### Ethics statement

Forest owners provided us with the permission to access the area and approved collecting of ground-dwelling spiders by utilized sampling method (pitfall trapping). The whole study was conducted under permit of Nature Conservation Agency of the Czech Republic (75/KK/2009) for Vladimír Hula in the frame of species monitoring under Natura 2000 (during 2009–2013). Collected data were transmitted for Nature Conservation Agency of the Czech Republic for using them in Nature Conservation Authorities. The study complied with all relevant national regulations. The voucher specimens are deposited in the collection of the first author at the Faculty of Forestry and Wood Technology, Mendel University in Brno, Czech Republic.

### Canopy openness evaluation

The light volume gradient was calculated using imaging software (GAP Light Analyzer, version 2.0) to extract canopy structural parameters and gap light transmission indices from true-color fisheye photographs [29]. This method obtains large-scale fractional canopy element cover and openness measures using hemispherical photography that can accurately capture the amount of light transmitted from canopy cover [52]. All hemispherical fisheye photographs were taken from ground level around the pitfall traps during one day to rule out seasonal effects (14 July 2012). The date was selected because the canopy is at its densest during this period (S1 Table).

### Spider species classification

All adult spiders were determined to species level in accordance with Roberts [50], Heimer & Nentwig [53], and Nentwig et al. [54]. Next, we classified the species for their conservation importance and usefulness for evaluating formerly coppiced forests. A straightforward process for evaluating the conservation and rareness values of the sampled species would be to compare their rarity and/or threatened status, which has proven very advantageous in many studies with conservation aspects [55–59].

We used degree of rareness, which was assessed according to Buchar & Růžička [51] and Růžička & Buchar [60], where classification of all species was in accord with their occurrence in the Czech Republic, thus as VR (very rare), R (rare), S (scarce), A (abundant), and VA (very abundant). In addition to degree of rareness, we analyzed the conservation value of the spider communities based on the Red List of Threatened Species in the Czech Republic [61] using the following categories: CR (critically endangered), EN (endangered), VU (vulnerable), and LC (least concern). We further used the abundance of rare species, which was calculated from the very rare, rare, and scarce categories, thus excluding abundant and common spider species [51,60].

In order to determine how the different “light environments” affect spider assemblages along the canopy openness gradient, we classified the spiders into three groups: open habitat specialist (OS), open habitat generalist (OG), and forest habitat species (FO). The classification is based on their requirements for light and humidity and their habitat preferences as quantified by Buchar & Růžička [51] and Kasal & Kaláb [62].

We also investigated the distribution of spiders’ hunting strategies along the canopy openness gradient. We used the hunting strategies proposed by Cardoso et al. [46] based on such functional traits as web usage or active hunting, type of web, occurrence in vegetation strata, daily activity, and trophic niche width. The hunting strategies are sensing-web weavers, sheet-web weavers, space-web weavers, orb-web weavers, ambush hunters, other hunters, ground hunters, and specialists [46].

### Statistical analyses

We investigated the relationships between the canopy openness gradient and spiders’ species richness, activity density, functional diversity, species composition, conservation value, and degree of rareness. All analyses were performed within the R environment [63] and Canoco 5 [64]. We estimated species richness and functional diversity per trap by individual-based rarefaction using the R package “BAT” [65]. To compute functional diversity, we used the functional traits connected to spiders’ hunting strategies proposed by Cardoso et al. [46]. We also incorporated spider size as a functional trait. Spider sizes were taken from Nentwig et al. [54]. We estimated the functional diversity using a dendrogram-based approach whereby the unweighted pair group method with arithmetic mean (UPGMA) was used as the agglomerating algorithm and Gower distance as the distance measure [66]. Estimation of both species richness and functional diversity was performed using 1,000 permutations, and the number of individuals was set at the lowest number of collected individuals (N = 10). We then used the mean value from the 1,000 permutations to study the relationships between canopy openness and species richness and functional diversity.

To calculate conservation value, the numbers of records of individual species in a sample were weighted using a ranked scale based on their presence in the Red List of Threatened Species in the Czech Republic [61] with different conservation statuses (CR = 4, EN = 3, VU = 2, LC = 1). We then calculated the degree of rareness according to Buchar & Růžička [51] and Růžička & Buchar [60]. In this evaluation, the numbers of records of individual species were weighted and ranked according to the occurrence and faunistic monitoring of species in the Czech Republic (VR = 5, R = 4, S = 3, A = 2, VA = 1).

We investigated the relationships between canopy openness and the variables using generalized estimating equations (GEEs) within the R package “geepack” [67]. The use of GEEs is a method that serves as an extension of generalized linear models for data with pseudoreplications [68]. We used an autoregressive correlation structure inasmuch as the traps within a location were distributed in regular intervals [68]. To investigate the degrees of rareness, conservation values, activity density, and abundances of rare species, we used GEEs with a Poisson error structure and log link (GEEs-p) because the data were counts [69]. The patterns of species richness and functional diversity were studied using GEEs with normal distributions of error (GEEs-gau) inasmuch as they became normally distributed after the rarefaction procedure [69]. The models’ linear predictor was of quadratic regression type because the relationship between canopy openness and the dependent variables could be hump-shaped due, for example, to an ecotone effect.

We studied the change in species composition along the canopy openness gradient using partial canonical correspondence analysis (CCA), where location acted as a covariate. We used CCA because the initial detrended correspondence analysis showed the gradient to be long (SD = 3). We downweighted rare species because CCA is sensitive to their presence [70]. We tested the significance of the canopy openness using Monte Carlo permutation tests (1,000 permutations) while restricting the permutations within the locations [70]. To investigate how habitat preferences and hunting strategies affected spiders’ distributions along the canopy openness gradient, we passively projected the traits of species into CCA biplots.

## Results

### Overview

We collected 1,945 adult spiders representing 20 families, 53 genera, and 90 species. Of these, 31 species were classified as having a preference for forest habitats and 54 species were classified as having a preference for open habitats with a higher level of canopy openness. More than one-third of all the species are known to be xerothermophilous with ecological restrictions to open and partly shaded habitats such as forest-steppe and sparse forests (N = 38). The records contained a total of 23 species (26%) listed in the Red List of Threatened Species in the Czech Republic [61]. In general, we discovered a substantially diversified spider assemblage with a large presence of rare species characteristic for open and xeric habitats (for species list, abundances, functional traits, conservation status, and degree of rareness, see S1 and S2 Tables).

### Species richness and functional diversity

The peak of species richness occurred in the middle of the canopy openness gradient along with symmetric decreases toward both extremes, thus suggesting an ecotone effect (GEE-gau, quadratic term, *χ*^*2*^_1_ = 27.5, *P* < 0.001, Fig 2a). Functional diversity showed a hump-shaped relationship, with the peak shifted more toward closed canopy and then a rapid decrease with increasing canopy openness toward open habitat structures in forest stands (GEE-gau, quadratic term, *χ*^*2*^_1_ = 9, *P* = 0.003, Fig 2b).

### Activity density of spiders

Spider activity density evinced a more or less asymptotic relationship with the asymptote very close to the completely open canopy in forest clearings (GEE-p, quadratic term, *χ*^*2*^_1_ = 5.1, *P* = 0.025, Fig 2c).

### Conservation concern

Abundance of rare and threatened species (GEE-p, quadratic term, *χ*^*2*^_1_ = 10.4, *P* < 0.001, Fig 2d), conservation value (GEE-p, quadratic term, *χ*^*2*^_1_ = 9.8, *P* < 0.002, Fig 2e) and the degree of rareness (GEE-p, quadratic term, *χ*^*2*^_1_ = 36.4, *P* < 0.001, Fig 2f) showed hump-shaped relationships with canopy openness. Their peaks were shifted toward more open canopy, where there were habitat structures with a high level of light volume.

### Spider community composition

**Fig 3:**
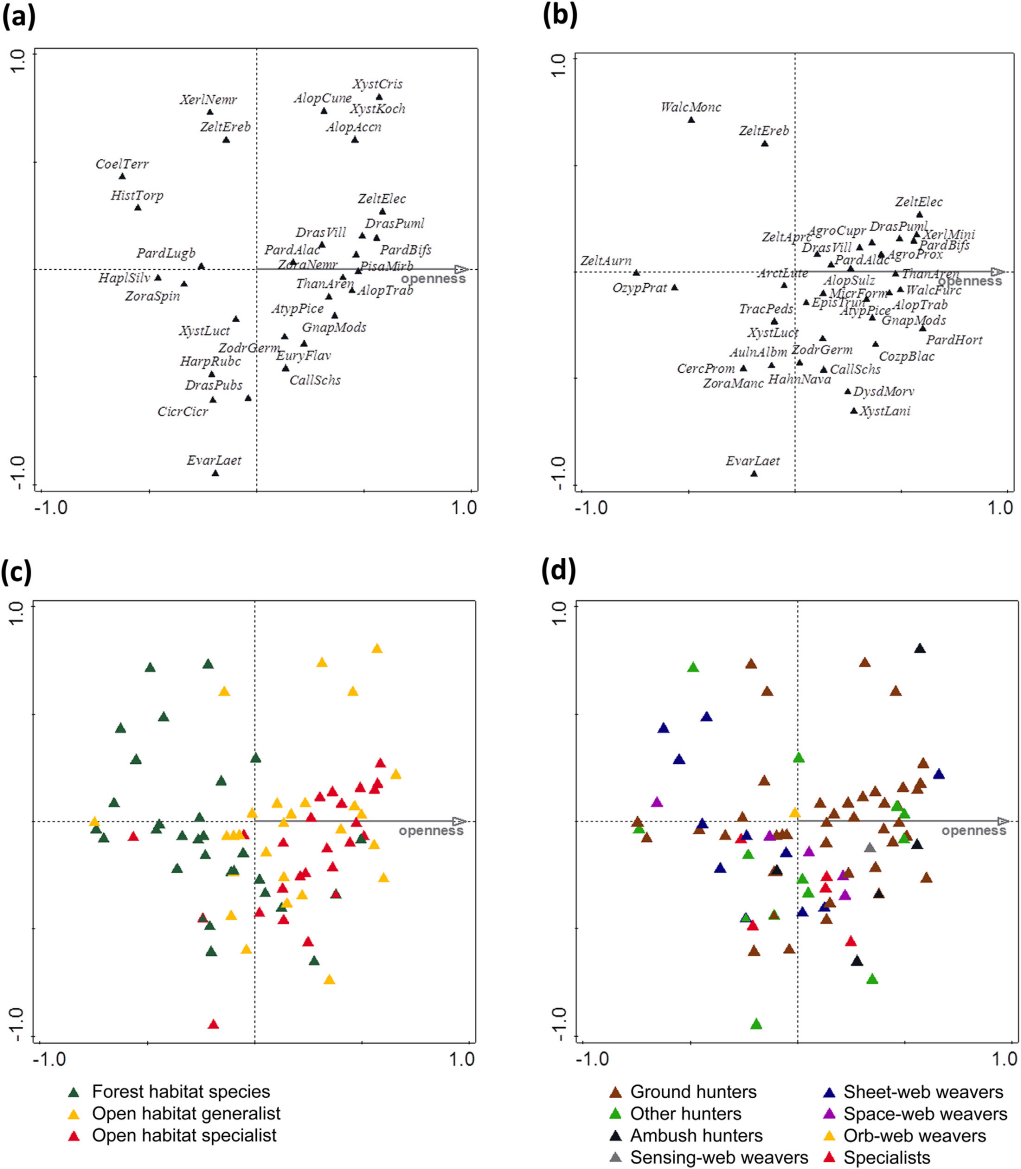
Biplot diagrams for canonical correspondence analysis (CCA) summarizing the differences in spider assemblages along the canopy openness gradient: a) thirty spider species, best fitted by canopy openness gradient (the canopy openness explained of overall variability 11.2%), b) only species of conservation concern, c) different ecological groups of spiders, d) spiders’ hunting strategies. The first three letters from both genus and species were used as an abbreviation in (a) and (b) graphs (see appendices for species list with full names).

Canopy openness significantly affected the composition of spider communities (CCA, pseudo-*F* = 3.9, *P* = 0.001, Fig 3a). The constrained axis explained the most variation (i.e., 11.2%). The first and second unconstrained axes explained 7.9% and 7.4%, respectively. Along such a short distance, there was rapid species turnover according to the species’ environmental niches as well as hunting strategies. Shaded conditions were preferred mostly by forest species. Most open habitat generalists had optima in moderately and more open canopy while open habitat specialists had optima mostly in more open and sparse canopy (Fig 3c). With respect to hunting strategies, web weaving spiders (and mainly sheet-web weavers) had optima in more closed canopy. There was only one web weaver that had its optimum in more open canopy (*Cercidia prominens* Westring, 1851). The optima of hunters were more evenly distributed along the whole gradient. Although ground hunters were distributed along the entire gradient, most ground hunters had optima in more open canopy. Consequently, the most diverse mixture of hunting strategies was under more closed canopy (Fig 3d). Most conservationally important species had their optima in more open and sparse canopy, but some had optima in the closed canopy of dense forest habitats (Fig 3b).

## Discussion

In the present study, we analyzed the impact of diverse light conditions on epigeal spider communities across the canopy openness gradient within one of the major areas of formerly coppiced oak forests in the Czech Republic. Although based on just small spatial patches of forest habitats, our results clearly show that species richness, functional diversity, activity density, and indicators of conservation concern were strongly influenced by canopy openness and had peaks at different levels of light conditions. In general, we found that formerly coppiced oak forests are species rich and important habitats for many rare and threatened spiders in intensified, modern landscapes of the Czech Republic. Almost 10% of the total 875 species known in the Czech Republic were recorded [71]. Furthermore, 26% of all species listed in the Red List of Threatened Species in the Czech Republic [61] and nearly 40% of regionally significant species that are among the rare to scarce bioindicators of the xeric (open to partly shaded) habitats were described [51,60]. According to our results, we showed that many spider species forming different ecological groups distinctly differed in their distribution between gradient of canopy openness even in the small spatial scale patches along forest ecosystem (60 m long transect). We found that the preferences of a considerable proportion of such conservationally important species as open habitat specialists (e.g., *Atypus piceus* [Sulzer, 1776], *Drassyllus pumilus* [C.L.Koch, 1839], *Gnaphosa modestior* Kulczynski, 1897, *Thanatus arenarius* L. Koch, 1872) had a strong linkage to early and mid-successional forest stages. On the other hand, more dense habitats with closed canopies (late succession development) were also important for some species of conservation concern dependent on shady and more humid conditions (e.g., *Ozyptila praticola* [C. L. Koch, 1837], *Cozyptila blackwalli* [Simon, 1875], *Walckenaeria monoceros* [Wider, 1834]) as well as for common forest species [e.g. *Coelotes terrestris* (Linnaeus, 1758), *Haplodrassus silvestris* (Blackwall, 1833)]. It is thus clear from our results that rapid and strong species turnover in a such short distance highlight the importance of habitat complexity for spiders within differing levels of canopy coverage. These results are in accord with findings by Oxbrough et al. [38] and Muff et al. [41] that showed importance of habitat variability for spiders even in a small spatial scales in forest plantations and alpine timberlines.

### Relationship between canopy openness and diversity indicators

The studied conservation indicators showed a hump-shaped relationship, with rising canopy openness confirming the importance of diversification in terms of more open and light conditions [16]. The optimal values of canopy openness for rare and endangered species of spiders were in the range of 65–75%, which corresponds to the characteristics of open and sparse lowland woodlands. Such woodlands are among the most diverse and biologically richest habitats of the temperate zone [1,17]. Oak woodlands have naturally higher canopy openness [72] due to the requirements of the dominant tree species (*Quercus* sp.). Moreover, oak woodlands in Central Europe were managed for a long time and associated species have adapted to more light and diverse conditions [4–8]. Inasmuch as open places in the forests with sparse canopies contained mature trees, shrubs, tall and short turf grasses, and small patches of bare soil, they created a mosaic-like combination of different microhabitats of early and mid-succession forest stages [6,11]. From this point of view, the mosaic-like combination of microhabitats creating highly structured and diverse habitat seems to produce conditions suitable for rare and endangered spider species. This pattern has been shown in numerous studies focusing on various forest and non-forest ecosystems [57–59,73–75]. Furthermore, the greater structural diversity in open forest can host more prey, more hideouts from unfavorable environmental conditions and enemies, and more living space [76]. The greater structural diversity can therefore increase the overall abundance of predaceous arthropods, as it was found that the peak of activity density of all spider species was at 90% of canopy openness. Moreover, spiders often display lower activity in dense forest. This most likely is due to the lack of herb layers, because even many ground-dwelling spiders use vegetation for hunting or nesting [51,77]. The overall decrease toward completely open canopy without the presence of trees standards probably was caused by exposure to such extreme environmental conditions as drought, high temperatures, lack of herb vegetation, as well as strong disturbance from large-scale thinning and logging of trees. Such conditions are unsuitable for spiders that require at least some partly shaded microhabitats with higher moisture and the presence of hideouts [37,41,74].

The peak of species richness occurred in the middle of the canopy openness gradient while there were symmetrical decreases toward both habitat extremes, thus suggesting an ecotone effect (changeover from open to more dense forest). The ecotone effect’s positive influence on species richness in forest habitats is well documented and proves the positive impact on species richness/diversity in forest habitats [78–80]. In our study locations, habitat structures in the changeover from open stands to dense stands included spider species expanding from both extremes but likely maintaining a suitable environment only for common and euryvalent species, while the presence of conservationally important species rose by a significant 65% with higher canopy openness.

In contrast to conservation indicators, the peak of functional diversity was shifted to more closed canopy (40%), albeit still in relatively open forest, although with the presence of more shaded microhabitats and higher litter layers typical for late succession stages of forest development [1]. The greater functional diversity in the more closed canopy occurred because many web-weaving spiders, and especially sheet-web weavers, had optima in more closed canopy. The optima of hunters were distributed more evenly along the gradient. Consequently, the mixture of hunting strategies was more diverse under more closed canopy than it was under open canopy. Web-weaving spiders are limited by the number of attachment points for their webs [81–83]. The number and diversity of attachment points for the various types of webs was highest at this level of canopy openness (40%) probably due to the presence of more litter layers and the most complex vertical stratification of vegetation layers. Herbs and shrubs, which are important for many orb-web and space-web weavers, decrease toward completely closed canopy [81,83] while tree standards and litter layer, which are significant for many sheet-web weavers, decline toward the more open canopy [84,85]. Completely open canopy provides the fewest attachment points because only a sparse herb layer is present in this habitat. Moreover, spider size, which we also used as a functional trait, is conserved within congeners [39]. Many congeneric spider species had optima in more open canopy while species that had their optima in more closed canopy were mostly heterogeneric. Thus, the lower proportion of congeners could also add to the higher functional diversity in more closed canopies of forest habitats.

### Implications for forest management

Most European oak forests were coppiced over centuries of traditional woodland management at a small spatial scale under the former historical ownerships prior to the Second World War [7,8]. All of these features contributed to desirable diversification of various microhabitats under different age rotations and logging disturbances [4,6]. Later, the abandonment of traditional methods, overall landscape changes, and transformation to old forest occurred within the state holdings created due to political changes. These changes were characterized by two distinctive extremes, both causing strong unification of canopy coverage followed by biodiversity loss in forest ecosystems [1,16]: 1) heavy logging and increasing disturbances, and 2) no active management and no disturbances. As demonstrated by our results and as confirmed by other studies, none of these management approaches constitute suitable tools for improving forest biodiversity in lowland oak woodlands. Furthermore, we conclude that it is not necessary to return to coppice management on the entire area of formerly coppiced forests, as has been suggested by some authors (e.g., [22,86,87]). This kind of management can bring unification of forest habitats in another form. It is rejected by foresters for economic reasons and even despite the fact that demand for fuel and firewood has been increasing in recent years due to European policy in support of renewable energy resources [88]. The traditional method of coppice management and forest structure still exist in most Balkan countries as well as in Austria and France [4,89,90]. In these countries, some species, usually termed “light forest species” or “coppice species,” are still locally common [90]. These include, for example, the butterflies *Euphydryas maturna* (Linnaeus, 1758), *Clossiana euphrosyne* (Linnaeus, 1758), and *Parnassius mnemosyne* (Linnaeus, 1758) [19]. There are no existing homogenous areas with the same age or rotation in forests, but there always are mixtures of different management types (beyond coppicing) with different patch sizes. Based on this knowledge and our results, we can state that activities in lowland oak woodlands should include only small-scale disturbances (e.g., small-scale timber harvesting) in order to support the differential values of successional stages required by various ecological groups of spiders or other arthropods [4,11]. The early and mid-successional stages, respectively, are important for rare and threatened species (open habitat specialists) connected with unmanaged forest patches of mature trees with more dense and shady conditions suitable for forest as well as some conservationally important species [91–93]. We propose at least such minimal interventions as conservation thinning, selective timber harvesting, and especially returning to private land ownership on small spatial scale patches in order to enhance coppice management in various age rotations (from early to late succession development). This would lead to restoring diversification of canopy openness and light conditions in formerly coppiced forests, thereby enhancing overall habitat complexity ranging from open to dense forest structures [11,16,93]. We suppose that these kinds of active interventions should be also used as a target management for some “coppice” species typical for open and sparse forest that usually did not abundantly occur in other habitats (e.g. *Atypus piceus*). Our results and all recent records from Moravia shows [11], that *A*. *piceus* prefers mainly open and light forest stands dry forest fringes. This species is among the one of the flagship species for conservation activities and nature protection efforts aimed on araneocenosis in the condition of the Central Europe. [94]. Interesting fact is, that our records are in contrary with data considering impact of disturbances [94]–we do not suppose that *A*. *piceus* cannot survive disturbances, however in the opposite way, it needs some light within the whole forest habitats, which is made by disturbances of canopy coverage only (e.g. conservation thinning, random timber harvesting). We conclude that these findings would be used in conservation managements considering presence of this species in habitats of lowland oak forests.

Within forest management, selective timber harvesting and thinning is often used as a conservation tool, particularly in tropical forests [95] but recently also in European forests [96]. Its positive effects on biodiversity have been reported [1,10]. In the case of state forests, mainly in protected areas (Natura 2000 sites, nature reserves, national parks, and others), the only way is to copy forests’ previous ownership structure based on old forest maps, all of which are available, or to establish new land-use schemes with a small-scale structure. A question is whether the Czech Republic will manage the forests in accordance with principles of nature conservation in view of the active management presently occurring in the floodplain forests of the Lower Morava River [17,24] or in thermophilic oak woodlands in Podyjí National Park [10]. We assume that active, artificial interventions in protected areas should be seen not as a threat to the forest environment but as an acceptable substitute for such traditional forestry practices as coppicing, pasturing, and natural disturbances (fires, windstorms, grazing by large herbivores) which no longer are occurring but which historically sustained a high level of biodiversity [2,3,7].

## Conclusions

Our study shows that the various indicators of spider diversity peaked at different levels of canopy openness in forest stands selected within the main part of South Moravia’s formerly coppiced woodlands. Most conservationally valuable species preferred more open canopy, but there were also a few that preferred more closed canopy. Species richness peaked in the middle of the gradient, while the peak of functional diversity was shifted more toward closed canopy. Moreover, there was a rapid turnover of spider species along the entire canopy openness gradient (which means depending upon their environmental requirements and hunting strategies). Rapid species turnover took place even on such small spatial scale as that represented by 60 m transects. This community change in such remarkably short distance highlights the importance of habitat complexity even on limited patches of forest stands. As habitat heterogeneity appears to play such a crucial role in the diversity of local spider assemblages, small-scale heterogeneity should be maintained in managing lowland oak woodlands. Based on our findings and with a view to spiders’ overall diversity, the scale relevant for creating benefits should be set at merely dozens of meters. We suppose that all the suggested improvements would promote species diversity, conservation aspects, and functional diversity. These are essential to ecosystem functions and the restoration of forest environments in landscapes under intense human land use.

## Supporting Information

S1 Table Total abundances and species density with values of canopy openness in studied plots (%). (XLS)

S2 Table Complete list of recorded spider species with abundances, functional traits, conservation status, and degree of rareness according to relevant literature. (XLS)
